# On the Role of Hollow Aluminium Oxide Microballoons during Machining of AZ31 Magnesium Syntactic Foam

**DOI:** 10.3390/ma13163534

**Published:** 2020-08-11

**Authors:** Sathish Kannan, Salman Pervaiz, Abdulla Alhourani, Robert J. Klassen, Rajiv Selvam, Meysam Haghshenas

**Affiliations:** 1 Department of Mechanical Engineering, American University of Sharjah, Sharjah 26666, UAE; skannan@aus.edu (S.K.); b00057903@alumni.aus.edu (A.A.); 2 Department of Mechanical and Industrial Engineering, Rochester Institute of Technology—Dubai Campus, Dubai 341055, UAE; 3 Department of Mechanical Engineering, Western University, London, ON N6A 5B9, Canada; rjklasse@uwo.ca; 4 Department of Mechanical Engineering, Manipal Academy of Higher Education—Dubai Campus, Dubai 345050, UAE; rajiv.selvam@manipaldubai.com; 5 Department of Mechanical, Industrial and Manufacturing Engineering, University of Toledo, Toledo, OH 43606, USA; Meysam.Haghshenas@utoledo.edu

**Keywords:** magnesium, alumina, microballoons, cutting

## Abstract

The role played by hollow ceramic thin-walled aluminium oxide microballoons on the shear deformation characteristics of AZ31 Magnesium syntactic foam is studied through high-speed machining. The ceramic microballoons embedded in the AZ31 matrix provides the necessary stiffness for these novel foams. The effect of hollow ceramic microballoon properties, such as the volume fraction, thin wall thickness to diameter ratio, and microballoon diameter, profoundly affects the chip formation. A novel force model has been proposed to explain the causes of variation in cutting forces during chip formation. The results showed an increase in machining forces during cutting AZ31 foams dispersed with higher volume fraction and finer microballoons. At a lower (Davg/h) ratio, the mode of microballoon deformation was a combination of bubble burst and fracture through an effective load transfer mechanism with the plastic AZ31 Mg matrix. The developed force model explained the key role played by AZ31 matrix/alumina microballoon on tool surface friction and showed a better agreement with measured machining forces.

## 1. Introduction

Metal matrix closed cell syntactic foams comprise of hollow alumina ceramic micro balloons dispersed into the AZ31 Magnesium (Mg) matrix through the squeeze casting method. In comparison to the traditional open-cell metal foams, these novel material systems offer a superior combination of specific strength, damping characteristics, and impact resistance [1,2,3]. For a variety of applications, in the industries of interest, and applicability, the chosen material needs to have good corrosion resistance, mechanical strength, and energy absorption capabilities under compression loading. Due to better specific strength and buoyancy of syntactic foam parts, they find potential applications in the manufacturing of submarine components, crashworthiness, and lightweight sandwich structures and biomedical applications such as temporary orthopedic bone implantations (plates, staples, screws, rods, and prosthesis) [4,5].

These requirements necessitated the interest to study the machinability characteristics of magnesium syntactic foams which are a relatively new class of materials to be shaped into useful technological products at lower manufacturing cost. Many studies have addressed the primary processing methods for metal syntactic foams such as casting [6,7,8]. Further, characterizations of their mechanical behavior upon impact or loading with various matrix and sphere materials have been carried out [9,10,11,12]. Several machining studies on porous cellular structures have been carried out. Face milling and grinding operations conducted on Titanium foams showed the significance of porous structure and its effect on machining associated surface defects and attainable surface quality [13]. The surface structure deviations introduced on aluminium open foam samples by machining-based processing and their effect on mechanical performance was studied [14]. The results showed an enhancement in foam mechanical performance through structural gradation. During the ultraprecision cutting of porous titanium, it has been observed that the cutting chip morphology was affected by the pore size and their location in the material. The crack initiation sites were at the pore locations and the tool wear was suppressed by the pores during wet cutting [15,16]. In their study on the precision machining of porous carbon, Heidari et al. [17] showed that porous carbon can be efficiently machined using diamond cutting tools. The feed rate and depth of cut were found to be the key process variables to control surface roughness. Studies on the surface integrity aspects of machining open-cell porous aluminium foams revealed the dependence of deformation affected machined layer on feed rate and axial depth of cut. An increasing trend in the size of the deformation zone by up to four times its original pore size is reported at a higher feed rate and lower cutting speed [18]. Qiao et al. [19] in their work used the micro-computed tomography to study machined surface integrity during face milling of open-cell aluminum metal foams. In their work, the characterization of effective pore size, foam porosity, and depth of the deformation-affected zone has been carried out. A reduction in pore size has been observed at the machined surface and the relative volumetric porosity has been shown to decrease significantly. Through their 2D mesoscopic finite element model, Guerra Silva et al. [20] attempted to analyze the chip formation process during machining cellular stainless-steel metals. The chip separation is initiated primarily due to tension loading of struts and followed by nodal shear loading. Studies on the micromachining characteristics of porous titanium samples showed a high level of interlink between the structural porosity of the material and the generated machining forces [21]. It has been shown by several studies that the machining of porous materials has been a challenging task primarily due to severe subsurface damage and smeared material caused as a result of chip tearing and heterogenous crack propagation during chip formation. Rafael et al. [22] have studied the interaction between the tool edge and the mesostructure during the peripheral milling of heat resistant austenitic stainless-steel cellular material. The chip formation and cutting forces were found to be highly dependent on the arrangement of cells.

**Figure 1:**
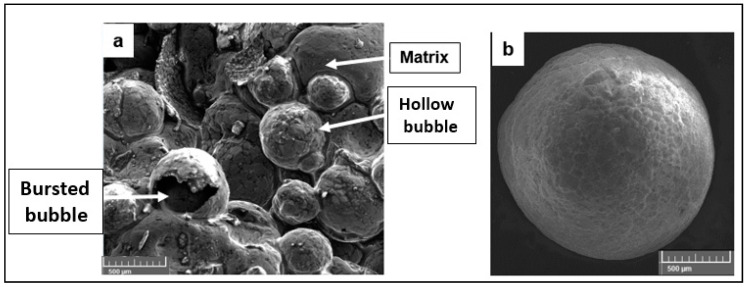

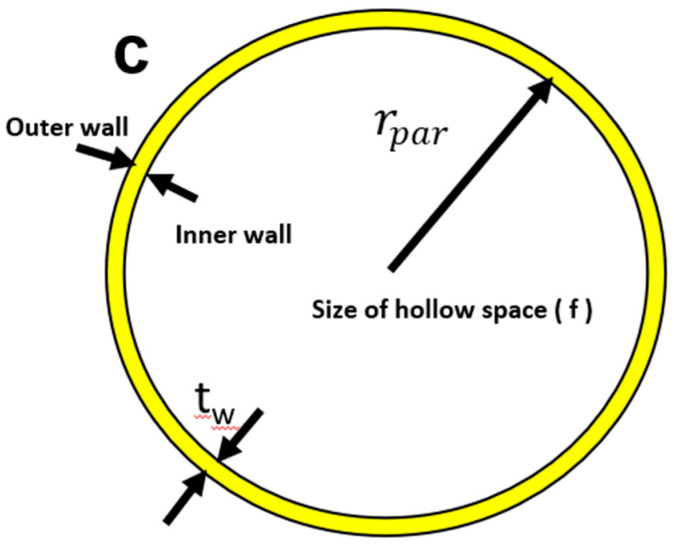
(**a**) A representative AZ31-Mg hollow alumina syntactic foam (**b**,**c**) hollow ceramic microballoon.

For a widespread application of these novel material systems and transforming them from their near-net shapes into a useful final product will require the appropriate development of machining methods. The technological products manufactured employing metal syntactic foams will require final conventional machining processing techniques, such as turning, drilling, tapping, reaming, and milling operations for making key features such as bolt holes, threads, screws, grooves, slots, etc. From the literature, it is evident that very few papers have been published related to the characterization of machining behavior of closed-cell metal syntactic foams. Investigation on the key deformation mechanisms constituting the chip formation during cutting AZ31 magnesium metal syntactic foams is necessary to tap the potential of these novel materials (Figure 1). It can be seen from the literature that most of the studies have focused primarily on the machining of open-cell porous structures. Also, there are no analytical or empirical models available in the literature for modeling the cutting behavior of magnesium-based metal foams. The availability of such models could be a very useful resource for furthering the knowledge on machining metal foams as the deviation in predictions from finite element models as a result of assumptions made on mechanical properties can be largely reduced.

In this paper, an attempt has been made to investigate the machining characteristics of AZ31 magnesium closed-cell foam materials. An attempt will be made to explain the mechanisms that constitute the machining chip formation through the development of a novel foam force model. The proposed model incorporates the characteristic plastic deformation behavior of AZ31 Magnesium matrix and its effective load transfer mechanism with hollow alumina microspheres. The effect of key factors such as microballoon wall thickness to average diameter ratio, bubble average size, and volume fraction dispersed into the AZ31 matrix on machining forces will be investigated.

## 2. Material

Hollow alumina microspheres were obtained from Pacific Rundum Co., Ltd. Tokyo, Japan and had an average bubble size ranging from 0.3–0.6 mm (data as provided by the supplier). These were cast into cylindrical aluminium syntactic foam billets for machining trials at Swamequip Ltd. India. The chemical composition and physical properties of AZ31 magnesium and hollow alumina bubbles (as provided by the supplier) used in this study are shown in Table 1 and Table 2. During the casting process, the stirrer RPM was maintained between 450–500 for 10 min. The temperature of the melt was 750 °C under inert ultra-high purity argon gas (3 L per minute) and the squeeze pressure was 117 MPa. The size of the castings was 50 mm diameter and 200 mm long. The electromagnetic vibrator was used at 300Hz to disperse the reinforcements into the melt. The mold was preheated to 300 °C and the hollow bubbles were preheated to 200 °C. The microstructure of AZ31 magnesium metal syntactic foam used in this study and the representative ceramic hollow bubble is shown in Figure 1.

### Constitutive Model for Closed-Cell Foam

Mechanical behavior of these metal foams has been characterized by several researchers in the past under various loading conditions [9,10,11,12]. The typical quasi-static deformation characteristic of AZ31 magnesium syntactic foam primarily comprises of a long linear elastic phase where stress and strain are directly proportional. This is followed by a sudden drop in stress which is considered as the yield point and initiation of plastic deformation of the matrix leading to a plateau phase. This reduction in stress is primarily due to the burst of the bubbles. During the plateau phase, the stress in the material is pretty much constant value accompanied by steadily increasing strain. This leads to the densification phase at which stage the bubbles have been burst and crushed to fill in the matrix void sites. In this phase, the Mg closed-cell foam shows deformation similar to a fully dense material as the stress increases steeply with increasing strain. The onset of yield phenomenon is important for estimating the mechanical properties of the Mg closed cell syntactic foams as the stress values attain their peak during the phase of the test. Typically, it is shown that the yield strength of metal foams decreases with increasing porosity [23]. The presence of hollow ceramic microspheres could significantly improve the load-bearing capability of the matrix through load transfer thus improving the yield strength of the AZ31 magnesium alloy foam. Equation (1) describes the of peak stress ($S_{max}$) of a magnesium alloy syntactic foam that is predicted using the yield strength of the magnesium matrix ($\chi_{y}$) and the area fractions of matrix ($R_{ma}$) and ceramic microsphere area fractions ($R_{CM}$) and is provided by [24].

(1) $$S_{max}=\left[2R_{ma}\chi_{y}+R_{CM}\Lambda Z_{f}\right]\left(\frac{O_{L}}{O_{C}}\;\right)$$

In the above expression, $Z_{f}$ describes the fracture strength of the hollow bubble wall. Ʌ takes into consideration of pores on the wall of the ceramic bubbles. The parameter Ʌ in the model assumed is 0.35 [24,25]. The hollow space size (f), is determined by the diameter of the sphere (Sd) and the thickness of the bubble wall, *t_w_*. The ratio $\frac{O_{L}}{O_{C}}$ that accounts for variation in shape of the bubbles and its effect on the shear stress can then be described using the ratio of bubble wall thickness to diameter, $\frac{t_{w}}{D_{avg}}$ as shown below [24]:

(2) $$\frac{O_{L}}{O_{C}}=1-\frac{f^{2}}{S_{d}^{2}}=4\left[\frac{t_{w}}{D_{avg}}-{\left(\frac{t_{w}}{D_{avg}}\right)}^{2}\right]$$

Uju et al. [26] proposed a method to determine the fracture strength of the hollow bubble wall, as follows:

(3) $$Z_{f}=\frac{E_{W}}{{\varsigma (1-Z_{R})}^{3/2}}$$

$E_{W}$ and $Z_{R}$ are the crushing strength and the void volume fraction in the hollow spheres and ς  is assumed to be 0.3 [25]. With the relative wall thickness ($t_{w}$/$r_{par}$), bubble radius $r_{par}$, and the microsphere volume fraction ($cm_{f}$), foam porosity is as shown below [27]:

(4) $$Z_{R}=cm_{f}{\left(1-\frac{t_{w}}{r_{par}}\right)}^{3}$$

## 3. Empirical Force Model for Machining Metal Syntactic Foams 

During metal cutting, the causes of force generation during cutting syntactic foams are due to (a) matrix hardening behavior and energy consumed for plastic deformation in the shear zone, (b) energy consumed to overcome friction in the secondary deformation zone due to chip sliding along the tool rake surface (c) other minor contributions such as plowing of the matrix material while cutting using a rounded cutting tool edge radius at lower values of feed (d) microsphere deformation characteristics such as the bubble bursting, debonding and crushing. Total force contribution can be shown as follows:

C_CF_ = C_p_ + C_f_ + C_pl_+ C_CR_+ C_D_ (5)

In Equation (5), C_CF_ is the total cutting force, C_p_ is the cutting force caused due to AZ31 Mg plastic deformation and matrix hardening, C_f_ is the force to overcome friction at the cutting tool-chip interface, tool plowing causes C_pl_, C_CR_ is caused due to the stress in the hollow ceramic microsphere leading to their burst and crush. C_D_ is the force due to the debonding of hollow bubbles from the AZ31 magnesium matrix as a result of interface failure. 

## 3.1. Force due to Plastic Deformation

The specific energy consumed for plastic deformation can be written as:

(6) $$SPL=\int_{0}^{\Omega }S_{max}\;d\Omega$$

where $S_{max}$ is the peak stress and dΩ is the incremental strain in the cutting zone. Substituting the expression which describes the plastic deformation of the closed-cell metal syntactic foam ($S_{max})\;$ from Equation (1) and modifying the Johnson–Cook model [28] leads to Equation (7).

(7) $$SPL=\int_{0}^{\Omega_{p}}\left[2R_{ma}\chi_{y}+R_{CM}\Lambda Z_{f}\right]\left(\frac{O_{L}}{O_{C}}\;\right)\left[1+Cln\dot{\varepsilon }\right]\left[1-{\left(\frac{T-T_{r}}{T_{m}-T_{r}}\right)}^{m}\right]\;d\Omega_{p}$$

Equation (7) is used to estimate the specific energy consumed during plastic deformation in the shear zone for metal syntactic foams in quasi-dynamic and dynamic conditions through the inclusion of strain, strain rate and temperature effects along with reinforcement properties such as volume fraction $\;\left(cm_{f}\right)$, average bubble size (Davg), (tw/Davg) ratio and crush strength of the hollow bubbles ($E_{W})$. The Johnson-Cook model parameters such as the shear strain, the shear strain rate, and the temperature in the shear zone can be determined by the relationships explained elsewhere [29]. The equivalent plastic strain, $\Omega_{p}$ during metal cutting can be obtained as a function of chip compression ratio (¶) [30]. By determining the specific energy spent for plastic deformation of AZ31 foam, the cutting force component ($C_{p}$) can be estimated using uncut chip thickness and chip width of cut.

(8) $$\Omega_{p}=1.15ln$$

## 3.2. Determination of Contact Friction between Microballoon/Matrix and Cutting Tool

Abrasion has been identified as the major contributor to tool wear during the machining metal matrix reinforced with ceramics [31]. The total friction force $\left(C_{f}\right)$ is considered to be contributed due to friction between cutting tool-ceramic bubble contact surface $\left(C_{CM}\right)$ and matrix–tool contact surface $\left(C_{m}\right)$.

(9) $$\left(C_{f}\right)=\left(C_{CM}\right)+\;\left(C_{m}\right)$$

The conditions of friction between the tool-magnesium matrix surfaces have been assumed to be similar to the pure metal as follows [32,33]:

(10) $$C_{m}=Sh_{avg}C_{cl}b_{tcw}$$

where $Sh_{avg}$ is the average shear stress at the tool-chip contact, $b_{tcw}$ is the true chip width, and $C_{cl}$ is the chip-tool contact length.$\;Sh_{avg}$ and $C_{cl}$ can be estimated using the ultimate tensile strength of the matrix material (St) and true undeformed chip thickness (*h_uc_*) as [33]:

(11) $$Sh_{avg}=S_{t}\;0.28$$

(12) $$C_{cl}=h_{uc}{¶}^{1.5}$$

Ceramic microballoons which are still intact in the matrix and did not burst during the shearing process engages in two body abrasion along with the hardened matrix and cause a material loss on the cutting tool rake surface. On the other hand, the fragments of the bubbles which have been busted and fractured along with debonded loose bubbles cause rolling friction between the hardened matrix and tool rake face causing three-body abrasion. The friction force contribution due to the ceramic hollow bubbles ($C_{CM})$ can be shown as:

(13) $$C_{CM}=C_{tb}+C_{thb}$$

where $C_{tb}$ represents the friction force due to two-body abrasion and is determined using several particles at the tool–chip interface $\left(N_{CM}\right)$, the contact area of the hollow sphere with the tool ($S_{a}$), yield strength of the tool ($S_{T_{O}}$) and Ï which is the probability of ceramic bubbles engaged in two-body abrasion (assumed 45%) as shown below:

(14) $$C_{tb}=N_{CM}S_{a}3S_{T_{O}}Ï\;$$

where $C_{thb}$ represents the friction force due caused due to the three-body rolling of the ceramic bubble debris as shown below [33,34].

(15) $$C_{thb}={\hat{u}}_{thb}\;n_{thb}$$

where ${\hat{u}}_{thb}$ is the coefficient of friction and $\;n_{thb}$ is the normal force due to three-body rolling abrasion. The method to estimate these factors is described elsewhere [29,34].

## 3.3. Estimation of Cutting Tool Ploughing Force

A rounded cutting tool edge is bound to plow through the workpiece causing material smearing, bubble cracking, and bubble dragging along the matrix material. The cutting tool edge was measured to be approximately 20% of the average size of the bubbles used in this study. The magnitude of this force can be approximated using the slip line field model and considering the matrix shear strength ($S_{h}$), tool edge radius ($C_{r}$) and cutting width ($c_{v}$) as described by Waldorf et al. [35]:

(16) $$C_{Pl}=S_{h}c_{v}C_{r}\tan\left(\frac{\pi }{4}+\frac{\gamma }{2}\right)$$

where $c_{v}=Cu_{O}\left[Y_{O}+\sin^{-1}\left(\frac{h}{Cu_{O}}\right)\right]+\frac{b-Cu_{O}\left(1-\cos Y_{O}\right)}{\sin Y_{O}}$, $Y_{O}$ is the approach angle, and $Cu_{O}$ is the nose radius. 

## 3.4. Hollow Ceramic Microsphere Burst, Crushing and Debonding

The following expressions are shown in Equations (17) and (18) from literature can be used to estimate the force due to ceramic bubble debonding, burst and crush from the AZ31 Mg matrix $\left(C_{D}\;and\;C_{CR}\right)$. The debonding force $\left(C_{D}\right)$ can be approximated using fracture energy per bubble (ʂ) along with undeformed chip thickness (h), the width of cut (b), and the number of bubbles in contact with the tool $\;(N_{CM}$). Ï is the fraction of bubbles that cause abrasion on the cutting tool. From calculations, it has been found that the magnitudes of these forces are negligible as shown by [34,36].

(17) $$C_{D}=ʂ\;bhN_{CM}Ï$$

(18) $$C_{CR}=\;E_{w}\pi t_{w}^{2}$$

## 4. Experimental Procedure

To measure the cutting forces for model validation, machining experiments were carried out using coated carbide cutting inserts obtained from Sandvik™ which had a 6° rake angle and 7° clearance angle. AZ31-Magnesium reinforced with hollow ceramic alumina bubbles with different volume fractions and average bubble sizes were used in the trials. All tests were conducted in a dry environment at different cutting parameters of uncut chip thickness (0.05, 0.1, 0.15, 0.2 mm), cutting speed (25 m/min, 50 m/min, 100 m/min), and reinforcement volume fractions (5%, 10%, and 15%) and average bubble sizes (0.3 and 0.6 mm) (Table 3). A KISTLER™ 9129AA three-channel dynamometer was used along with a multichannel charge amplifier type 5080 to measure the cutting forces. Chips were collected after each test to measure the shear angle. Tests were repeated and average values noted. Table 4 shows the required model constants for this study.

## 5. Results and Discussion

### 5.1. Deformation Mechanisms in the Primary Shear Zone 

#### 5.1.1. Effect of Cutting Speed

**Figure 2:**
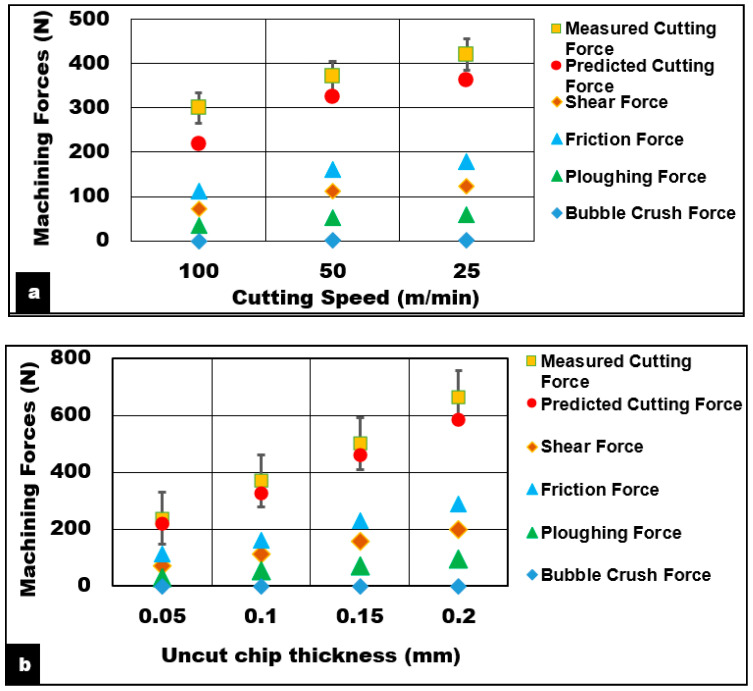
Effect of (**a**) cutting speed (**b**) uncut chip thickness on machining forces AZ31 Mg/hollow alumina syntactic foams (Volume fraction = 15%, h = 0.1 mm, b = 3 mm, Dry cut).

Effect of process parameters (speed and uncut chip thickness) on the variations in cutting forces during machining AZ31 magnesium foam reinforced with a 15% volume of alumina hollow bubbles is shown in Figure 2. The contribution of various mechanisms towards the total cutting force component is shown in detail. It can be seen from the results that an increase in cutting speed leads to a decrease in generated cutting forces by up to 120 N. However, the opposite was observed for changes in uncut chip thickness where the cutting forces showed an increasing trend. The friction force was found to be the major component of all cutting conditions tested in this study. The magnitude of friction force increased with increasing feed and decreasing cutting speed as shown in Figure 2.

The chip formation during cutting AZ31 foam at high cutting speed is influenced by an interconnected deformation mechanism associated with the plasticity of magnesium matrix and brittle thin-walled microballoon. Here, it becomes necessary to understand the interactive effects of matrix-microballoon load-transfer concerning shear zone strain rate and temperature. This correlation can be made with matrix plasticity in terms of twinning and prismatic/pyramidal slip processes. As the foam is subjected to high compressive force by the cutting tool, the presence of precipitate phases in the matrix such as γ-Mg17(Al,Zn)12 supports the load along the tool feed direction as the foam deforms elastically [37]. When a critical value of shear stress is reached, the shear strain in the magnesium matrix moves away from being elastic indicating the transfer of load to the elastic microballoons as the magnesium matrix starts deforming plastically. At this stage, the presence of microballoons shows their importance in load-bearing capacity by unloading the matrix. This load-bearing phenomenon has been shown in the literature [38,39].

As dynamic strain rate increases with cutting speed, the rate of twin nucleation, and subsequent increase in the volume fraction of twins in the magnesium matrix along the shear zone builds up. However, after a critical density of the twin population is reached, the plastic deformation in the matrix acts as an obstruction to the twin nucleation and growth via the grain boundaries [40,41]. Given the orientation of twins being less resistant to the prismatic slip leads to a transition from the dominant twinning mechanism to prismatic slip along the grain boundaries. The temperature dependence of critically resolved shear stresses of slip and twinning mechanisms in magnesium is well known [42]. The twinning and dislocation slip is characterized by a sharp drop in their critical resolved shear stress values that decrease drastically with temperature [42]. At higher cutting speeds, the material in front of the cutting tool begins to deform. Significant heat generated due to large shear deformation is concentrated into the shear zone and with very less heat conduction causes localization of heat in the cutting zone. The temperature in the cutting zone predicted from the model was in the range of 250–350 °C for the different cutting speeds considered. The concentrated heat generation in the shear zone aids in the annihilation of dislocation pile-ups along the twin boundaries which once acted as effective barriers of prismatic slip. The upper end of the cutting temperature range is close enough to cause transformation in the AZ31 microstructure, grain refinement, and dynamic recrystallization [43]. This changes the nature of stress distribution in the matrix and could potentially affect the load transfer mechanism through the interface causing adhesive cracks to initiate and propagate along the boundary. As a result of an interface failure, the micro balloons are debonded and pulled out of the matrix thus transferring the load back to the matrix. Following this, the matrix undergoes a severe shear deformation process as the hollow cavities left by the microballoons collapse. During this period, the shear stress falls drastically as the load-bearing capacity is diminished due to the absence of microballoons. This failure mechanism is proposed as a possible reason for the reduction in cutting forces with increasing speed during the machining of magnesium syntactic foam.

**Figure 3:**
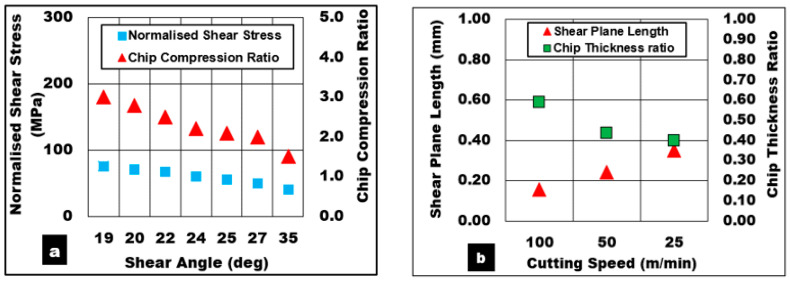

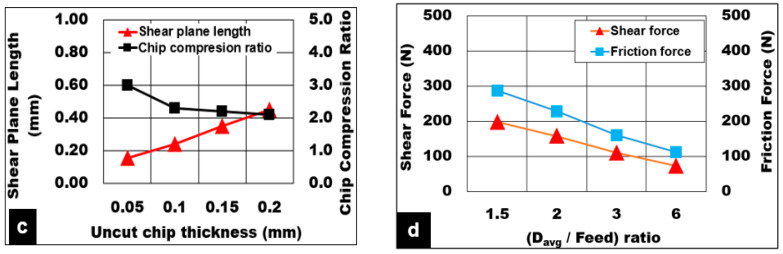
Relationship between various cutting parameters. (**a**) Effect of increasing shear angle on shear stress and chip compression ratio, (**b**) Effect of cutting speed on shear plane length and chip compression ratio, (feed = 0.1 mm). (**c**) Effect of uncut chip thickness on shear plane length and chip compression ratio (Vc = 50 m/min), (**d**) Correlation between (microballoon diameter to feed ratio) with machining forces. (Bubble volume fraction = 15%, b = 3 mm, Dry cut).

These findings are in line with the results shown in Figure 3a, where the normalized shear stress drops almost by a factor of 2 from 80 MPa to 39 MPa when the cutting speed (which is associated with strain rate factor) is increased from 25 m/min to 100 m/min accompanied by a rise in temperature along the shear zone. As seen in Figure 3b, a rise in cutting speed leads to a drop in chip thickness, hence increasing the chip thickness ratio with shortened shear plane length and steeper shear angles. As a result, a fall in shear stress required to separate the chip from the workpiece is also noted.

#### 5.1.2. Effect of Uncut Chip Thickness

Experimental and predicted results of the effect of undeformed chip thickness while cutting AZ31/15% syntactic foams are shown in Figure 2b. The measured cutting forces (Max: 585 N) and friction forces (Max: 282 N) increased with undeformed chip thickness up to 0.2 mm. This is primarily attributed to an increase in chip load. A further increase in feed is expected to cause a drop in machining force due to concentrated thermal softening of the matrix as observed during machining of AZ31 alloy. At lower uncut chip thickness values, the use of rounded cutting tool edge causes the phenomenon of tool rubbing and burnishing of the surface results in material smearing. This action raises the temperature (330 °C) in the shear zone. Higher material shear strain (1.26) is induced accompanied by a thinner chip (0.15 mm) and a reduction in chip thickness ratio (0.33). The effect of the chip thickness ratio on the shear angle is more pronounced. Reduction in the chip thickness ratio causes a shallow shear angle (19°) with a shortened shear plane length (0.15 mm). This is primarily due to a slower rate of chip flow (17 m/min) compared to cutting velocity (50 m/min) as it is separated from the workpiece. Shallow shear plane angle directly affects the rate at which chip flows. As the shear angle decreased from 26° to 19° with decreasing feed (0.2 mm to 0.05 mm), the chip compression ratio increased from 2.1 to 3 with decreasing chip flow velocity (24 m/min to 17 m/min). The model predictions for various conditions of uncut chip thickness were within 5% error for lower values of feed and increased to 12% of error margin as the feed increased up to 0.2 mm.

### 5.2. Effect of (Davg/h) Ratio

**Figure 4:**
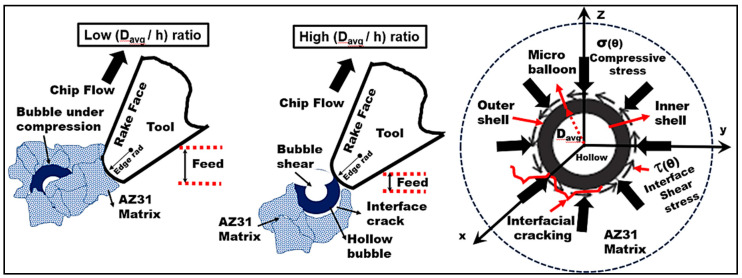
Effect of (Davg/h) ratio on hollow microballoon stress state during cutting.

To understand the role played by the bubble contact area with the tool, a correlation between the ratio of microbubble diameter to undeformed chip thickness (Davg/h) with shear force was established (Figure 3d). The average bubble size used in this study was approx. 0.3–0.6 mm. The range of (h) values used in the test was between 0.2 mm and 0.05 mm. It was noted that as the (Davg/h) ratio increased from 1.5 to 6, the magnitude of the primary shear force decreased almost by a factor of 2.75 (Figure 3d). It can be inferred that when the undeformed chip thickness (0.05 mm) is significantly smaller than the average bubble diameter (0.3 mm), the bubble contact area with the cutting edge in the shear zone is expected to be lower. The tool rubbing effect at lower uncut chip thickness values could cause surface smearing and most probably shear the bubble as the shear strength of the ceramic bubble is much lower than its compression strength. In this mode of bubble deformation, they were subjected to shear failure and the AZ31 matrix was exposed to the applied shear load (Figure 4). This furthered the rate of AZ31matrix plastic flow through the collapse of hollow cavities thus diminishing the effective load-bearing capacity of the microbubbles. In addition to the aforementioned ineffective load transfer mechanism, the higher rate of matrix plasticity is associated with cutting temperature in the primary shear zone. Moreover, at high (Davg/h) ratios, high cutting temperatures are estimated (350 °C) due to tool rubbing leading to higher values of equivalent plastic strain and chip compression ratio. It can be inferred through the literature findings [40,41] that the twinning mechanism which dominates the magnesium matrix plasticity during lower plastic strain is replaced by dislocation slip mechanisms (such as prismatic/pyramidal slip) at a higher equivalent plastic strain. This transition in magnesium plastic deformation from twinning to dislocation slip mechanism at lower cutting feed is expected to be associated with a drop in shear stress values with increasing cutting temperature in the primary shear zone (Figure 3d). 

However, at low (Davg/h) ratios, when the uncut chip thickness (h) was comparable or higher than the bubble diameter, the probability of contact area of the bubble with the tool cutting edge in the primary shear zone decreases. This means the microballoons ahead of the cutting tool and in the primary zone are subjected to almost all-sided compression which is more favorable to avoid bubble failure as they possess good compression strength. In this cutting mode, the bubble shear failure is minimized through an effective load transfer mechanism through the matrix and their precipitates which help to unload the microballoons. The bubble in this mode will carry the major share of compressive load until its burst event thus increasing the cutting force. Besides, the higher the undeformed chip thickness, the larger the chip load and volume of metal cut. This translates into a higher volume fraction of microbubbles in the shear zone that acts as an effective load-bearing medium thus enhancing the shear strength of the AZ31 matrix. This enhancement in the shear strength of the matrix is attributed to the high density of twins at lower equivalent plastic strain and its positive interaction with dislocation slips [41]. This mechanism which plays a major role in the matrix resistance to plastic deformation contributes to an increase in shear force required to form the chip at a higher cutting feed.

### 5.3. Deformation Mechanisms in the Secondary Shear Zone

**Figure 5:**
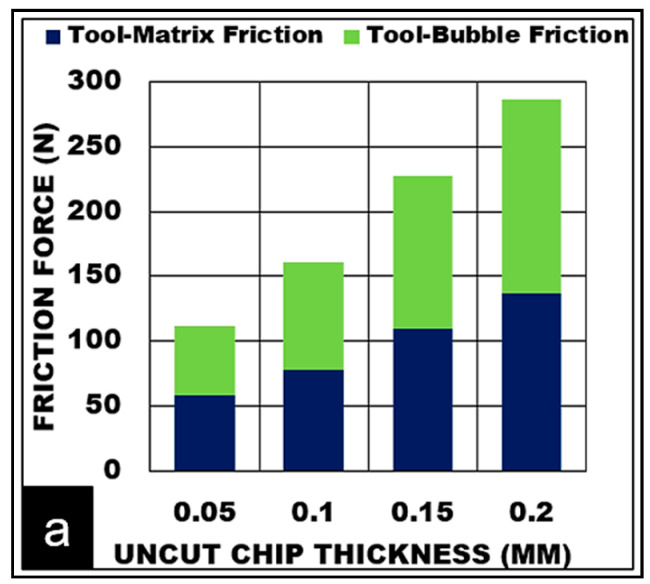

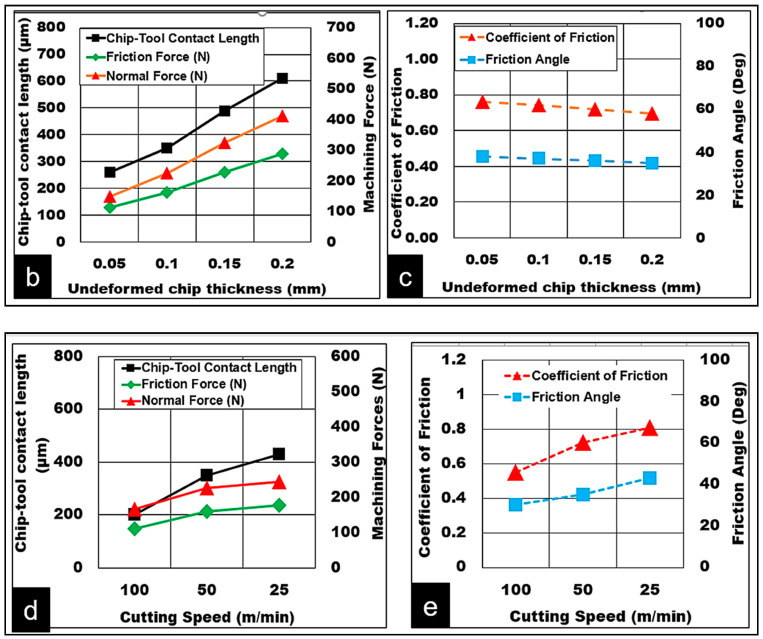
Relationship between various AZ31 foam cutting parameters (**a**) Friction due to the matrix and microballoon abrasion with increasing uncut chip thickness (**b**,**c**) Effect of uncut chip thickness on tool-chip contact length and friction coefficient (**d**,**e**) Effect of cutting speed on tool-chip contact length and friction coefficient, (Bubble volume fraction = 15%, b = 3 mm, Dry cut).

While cutting AZ31 metal matrix foam, deformation in the secondary shear zone is mainly influenced by tool-chip contact length which is dependent on cutting speed and uncut chip thickness. The force exerted by the tool on-chip arises primarily due to the normal force and the force to overcome interface friction. At this stage, the chip sliding along the rake face comprises of AZ31 matrix that undergoes severe plastic deformation in the secondary shear zone. Hollow cavities formed due to a fraction of microballoon pulled out gets collapsed and causes densification of the matrix. This phenomenon causes the tendency of plastic AZ31 magnesium to stick heavily on to the cutting tool edge as the chip thickness ratio increases and causes high normal forces in the secondary shear zone at a higher feed. In this process, a certain fraction of the micro balloons are embedded deep inside the soft matrix would stay intact, or in a burst or collapsed mode. These ceramic microballoons in combination with heavily deformed and densified matrix engage in two-body abrasion along the tool rake face and contribute to an increase in friction force. Dimples on the sliding surface also indicate the operation of three-body rolling abrasion due to fragments of collapsed ceramic bubbles. As can be seen from Figure 5a, as the cutting feed increases, the contribution towards friction forces due to matrix and bubble abrasion increases almost by a factor of 2.5. The chip tool contact length doubled (260 μm to 600 μm) with a change in uncut chip thickness from 0.05 mm to 0.2 mm causing a higher contact area of the chip in the secondary shear deformation zone. An Increase in chip flow velocity is also noticed from 17 m/min at the lower feed to 25 m/min at the higher feed. The friction force (112 N to 287 N) and normal force (147 N to 412 N) increased by approximately 40% even with a moderate reduction in friction coefficient and friction angle (Figure 5b). The degree of thickening of the chip (chip compression ratio) depends on chip-tool interaction and coefficient of friction. To reduce the chip compression ratio, a reduction in friction coefficient is necessary. The chip-tool contact shear stress in the secondary zone increased from (140 MPa to 160 MPa) with increasing cutting feed. This correlates with the phenomenon of severe matrix sticking to the tool edge during the densification process causes an increase in tool normal force. besides, mechanical abrasion on the tool rake face due to sliding of the hardened matrix along with the embedded ceramic hollow bubbles causes a subsequent drop in the chip compression ratio (3.0 to 2.1), friction coefficient (0.8 to 0.7), and friction angle (40° to 35°), resulting in a spike in both friction force and normal force (Figure 5c).

Figure 5d,e shows the effect of cutting speed on secondary shear zone deformation factors during machining AZ31 magnesium foam. It is noted that increasing cutting speed results in a significant increase in chip flow velocity (10 m/min to 60 m/min) accompanied by a shortened chip--tool contact length (430 μm to 200 μm). A 35–40% spike in frictional shear stress and normal stress on tool rake face is noticed with a corresponding reduction in chip compression ratio (2.5 to 1.7) with thin discontinuous chips at higher cutting speeds. Since the time for adhering microchips will be low, the tendency of the densified AZ31 matrix to form built-up-edge and sticking to the cutting edge is reduced at higher machining speed. A shorter chip–tool contact length means the number of matrix embedded bubbles in contact with the rake face decreases the tendency of two-body abrasion. This effect minimizes the spike in friction and normal forces thus lowering the friction angle (40° to 30°) and the coefficient of friction (0.8 to 0.5). Frictional conditions in the secondary shear zone are found to be more sensitive to changes in cutting speed compared to uncut chip thickness during cutting magnesium-ceramic hollow bubble foams. 

### 5.4. Mechanism of Chip Formation during Cutting Metal Syntactic Foam 

In cutting AZ31 magnesium foams, the discontinuous chip formation is dependent on factors such as the chip compression ratio, shear angle (ϕ), and matrix transverse cracking. At the start of the cutting process, the work material in front of the tool cutting edge is subjected to all-sided compression. The microballoons embedded in the matrix have good compressive strength. At this point during cutting, the matrix unloads the microballoons by carrying the load in an elastic deformation model. The γ-Mg17(Al,Zn)12 precipitates in the AZ31 matrix to a great extent promote an increase in stiffness of the foam by sharing a portion of the elastic load [37]. At lower plastic strains, the dominant strengthening mechanism in the AZ31 matrix is through nucleation of the high density of twins. The presence of twins is more favorable to enhance the mechanical strength of the material through their positive interaction with dislocation slip mechanisms acting as barriers to their motion in the twins and along the twin boundaries. This mechanism offers the effective load-bearing capacity of the magnesium foams. However, as the cutting progresses and material reaches closer to the tooltip, high shear stresses develop in the material and the deformation localizes into a narrow shear zone as the micro defect pile up continues along the twin and grain boundaries restricting their motion and increasing material strength. A significant degree of heat generation is caused due to the concentration of deformation in the shear zone. The twin volume fraction grows to a certain extent and cannot be limited anymore within the magnesium grains. With cutting temperature rising in the shear zone, this forces yielding of the magnesium matrix through a transition in deformation mechanism from mechanical twinning to dislocation slip [40]. At this stage, when the matrix starts deforming plastically, the bubbles carry the load through an effective load transfer via the interface, thus unloading the matrix.

The matrix deforms extensively causing defects and other slip mechanisms piling up along the interface with the microballoons causing severe stress build-up. The intensification of matrix-bubble interface stress initiates the coalescence of micro-cracks in the matrix that are cohesive. These matrix phase microcracks which form in the transverse direction to the feed direction are exacerbated due to high heat generation in the shear zone and merge with the longitudinal adhesive interface cracks.

**Figure 6:**
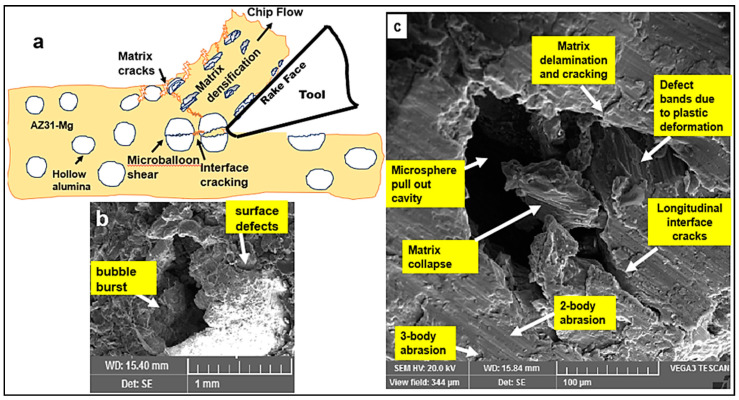
Ceramic microballoon failure mechanisms constituting shear failure during cutting AZ31 metal syntactic foams. (**a**) representative chip formation (**b**) fractured bubble (**c**) key deformation mechanisms

At this stage, there are three types of microballoon failure mechanisms that can potentially contribute to the continuation of crack propagation (Figure 6). The first mode of failure corresponds to the interfacial debonding of the microballoons causing microballoon pull out from the magnesium matrix leaving a hollow cavity. Alternatively, the interface cracking could embed the debonded bubble deep into the plastic magnesium matrix. The second mode of failure happens when the critical value of applied compressive force on the bubble surface initiates a bubble burst and fracture thus transferring effective load back to the matrix to further collapse the bubble cavities and take it into the densification phase through intense shear deformation. The third type of failure mechanism usually expected at higher values of average bubble diameter to feed ratio (Davg/h) which causes the bubbles to be sheared by the cutting tool as their shear strength is much lower than compressive strength, thus constituting a shear failure and crack propagation. 

Coalescence of high-density matrix transverse and interface longitudinal cracks happen along the matrix grain boundaries and bubble interfaces. The longitudinal adhesive cracks get aligned along the direction of maximum shear stress that minimizes cutting energy. They are initiated near the bubble interfaces and start to propagate from tooltip towards the free surface of the chip through effective crack propagation along with areas of maximum energy release rate to form discontinuous chips.

### 5.5. Effect of Microballoon Volume Fraction and Average Diameter 

**Figure 7:**
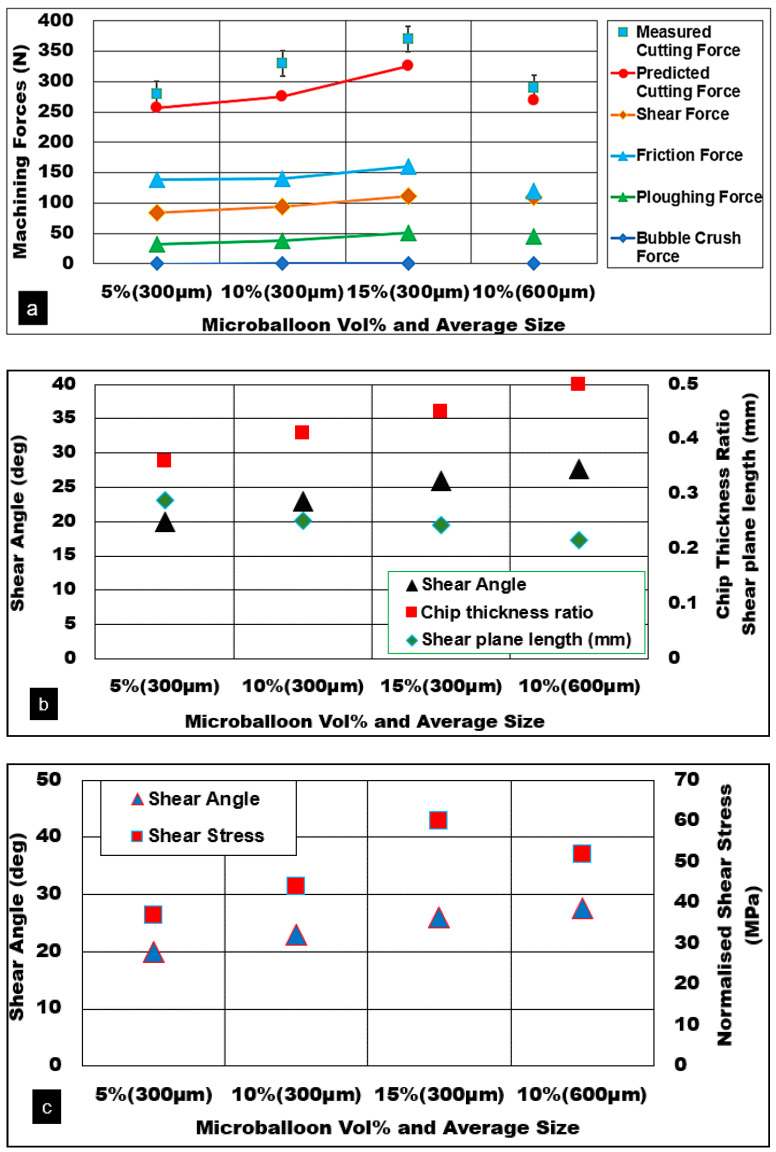
Cutting force predictions: effect of hollow bubble volume fraction and size AZ31 Mg/hollow alumina bubble syntactic foam, (h = 0.1 mm, Vc = 50 m/min, b = 3 mm). (**a**) Measured and predicted cutting forces (**b**) volume fraction vs shear angle (**c**) volume fraction vs normalized shear stress

The effect of ceramic hollow microballoon volume fraction and its average size on the cutting force and friction force is shown in Figure 7a. The presence of microballoons in the AZ31 matrix provides the required foam stiffness through an effective load transfer mechanism. An increase in the volume fraction of microballoons in the matrix improves the shear strength of the matrix through the effective pinning of the grain boundaries. Matrix plasticity is restricted due to the presence of a higher volume fraction of microballoons with the equivalent plastic strain reduced from 1.1 to 0.9. An increase in volume fraction means a higher number of microballoons in the primary shear zone. It is noted that as the volume fraction of ceramic bubbles goes up, the thermal conductivity and specific heat capacity properties are slightly reduced for the magnesium foam. This effect can be correlated with decreasing cutting temperature in the primary shear zone with increasing volume fraction. With lower mobility of dislocation slip mechanisms and reduced equivalent plastic strain, the shear angle increases from 20° to 26° with a corresponding increase in chip thickness ratio from 0.35 to 0.45 and normalized shear stress values (37 MPa to 60 MPa). Machining higher volume fraction foams with pronounced brittle behavior due to the presence of ceramic microballoons result in the formation of thinner discontinuous chips. The nature of chips formed shows the operation of active-matrix and interface crack propagation mechanisms where a reduction in length of the shear plane is predicted along with a substantial fall in the chip compression ratio (2.8 to 2.3) for higher volume fractions. 

As can be seen from Figure 7a, the measured cutting forces increased with volume fraction, however, they dropped with foams reinforced with coarser hollow bubbles. It is known that as bubble size becomes coarser, they possess a higher percentage of porosity and voids on their wall thickness hence causing the walls to burst/crack at relatively lower compressive forces compared to finer microballoons. As the cutting feed increases, the (Davg/h) ratio reduces. As a result, the plastic AZ31 matrix unloads the bubble and precipitates in the matrix help to share the load. Finer bubbles tend to be easily embedded inside the soft matrix on the application of all-sided compressive force under the rounded cutting tool edge compared to coarser bubbles. Due to their size factor and higher wall porosity concentration, magnesium foams reinforced with coarser ceramic microballoons are prone to shear damage or burst/fracture, leading to a reduction in cutting forces.

**Figure 8:**
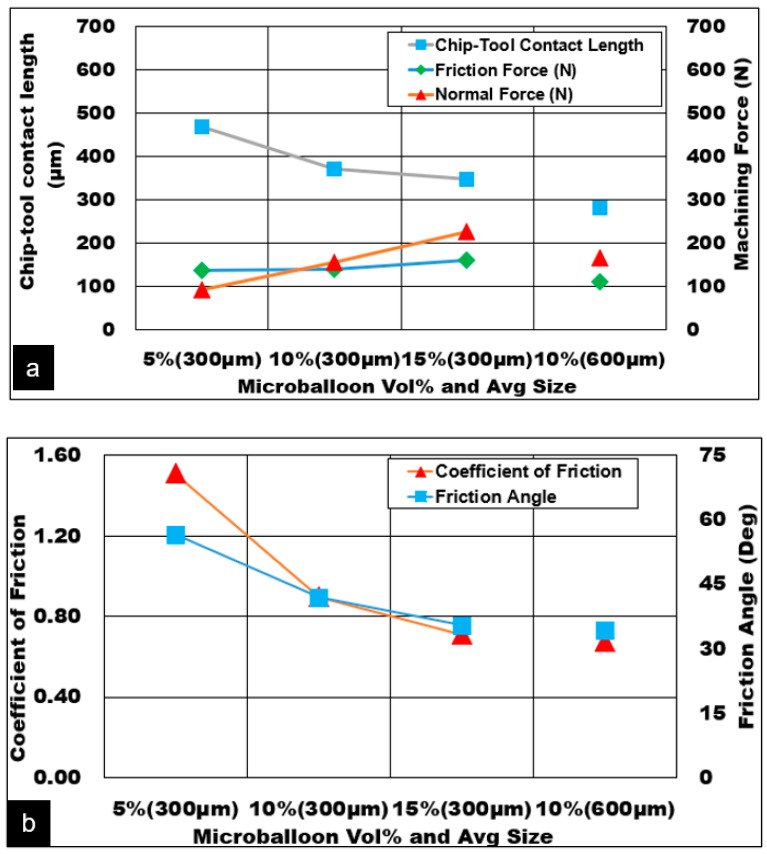
Friction force predictions: effect of hollow alumina vol% and average diameter. AZ31 Mg/hollow alumina bubble syntactic foam, (h = 0.1 mm, Vc = 50 m/min, b = 3 mm), (**a**) volume fraction vs chip tool contact length (**b**) volume fraction vs coefficient of friction

Figure 8 shows various mechanisms that contribute towards increasing friction force and normal force in the secondary shear zone. While dry cutting higher volume fraction foam, the normal force increased only by 40%, however, the friction force spiked by 86%. This clearly shows the role of plastic deformation mechanisms of the densified magnesium matrix aided by contributions from various load transfer mechanisms of ceramic microballoons discussed earlier. As can be seen, the chip-tool contact length decreases (470 μm to 350 μm) with increasing volume fraction. This shows the reduction in the plasticity of the matrix with higher predicted secondary zone shear stress values of 153 MPa. The adherence tendency of higher volume fraction magnesium foam to the tooltip is reduced with increasing chip flow velocity and reduced chip compression ratio.

Shortening of shear plane length and formation of discontinuous chips with increasing volume fraction can be attributed to the higher number of ceramic microballoons aligning along the primary shear plane. This increases the density of longitudinal crack nucleation sites and their accelerated propagation and coalescence with multiple crack sites along the primary shear zone resulting in a shorter chip-tool contact length. A reduction in the coefficient of friction and friction angle is noted. Machining at higher microballoon volume fractions halved the coefficient of friction (1.5 to 0.7) and reduced the friction angle by up to 20° (55° to 35°).

**Figure 9:**
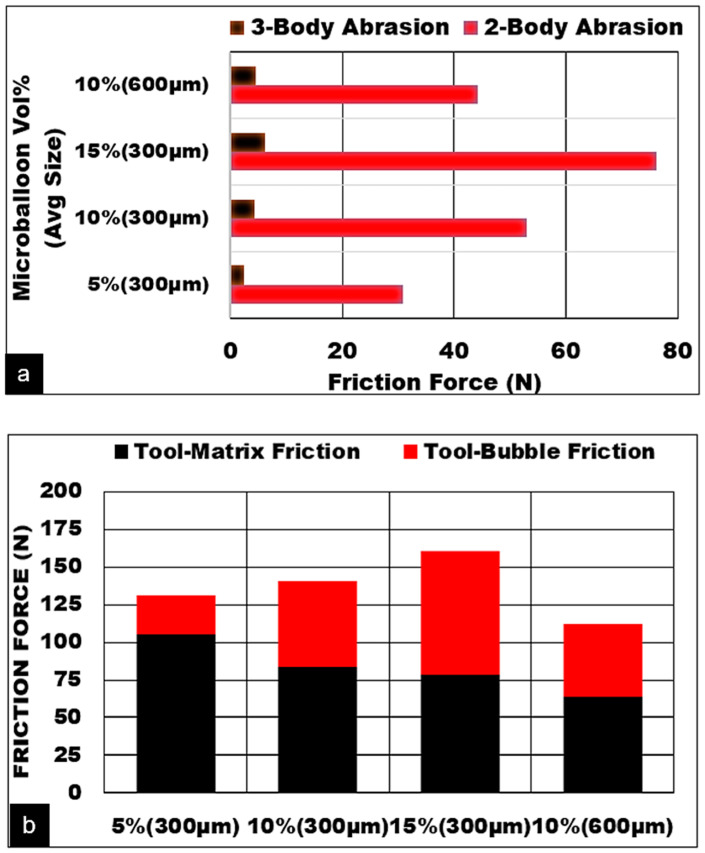
Contribution to friction forces (**a**) Abrasion mechanisms (**b**) Friction due to matrix and ceramic hollow alumina bubble syntactic foam, (h = 0.1 mm, Vc = 50 m/min, b = 3 mm, Dry cut).

The work-hardened matrix with a higher number of embedded bubbles engages in mechanical abrasion on the rake face. Finer bubbles along with second phase precipitates provide the required foam stiffness and cause the shear stress to increase in the secondary zone. As can be seen in Figure 9a, the contribution due to direct two-body abrasion of the cutting tool is much higher than three-body rolling. An increase in volume fraction makes it worse with a higher degree of two-body abrasions. The finer the ceramic bubbles, the higher the two-body abrasion. This is attributed to a higher degree of mechanical sliding of work-hardened magnesium matrix in combination with embedded ceramic bubbles against the rake face. With increasing volume fraction, the contribution to the magnitude of friction force due to ceramic bubbles increases exponentially (Figure 9b). The proposed model was able to predict the variations in friction force as a function of bubble size, bubble wall thickness to diameter ratio, and volume fraction of bubbles satisfactorily.

## 6. Conclusions

A constitutive force model for the machining of AZ31 magnesium syntactic foams reinforced with ceramic alumina micro balloons is presented. The developed model accounted for the hollow alumina microballoon reinforcement properties such as volume fraction, bubble shell wall thickness to diameter ratio, and the bubble crushing strength for closer prediction of cutting forces. It is shown that the presence of hollow ceramic microspheres in the AZ31 magnesium matrix influences the shear strength of the matrix through the characteristic load transfer mechanism. The following conclusions can be drawn from this work:

1. The higher the microballoon volume fraction and finer their average size, the higher the generated machining forces. Finer balloons improved the shear strength of the matrix, possibly through effective pinning of the grain boundaries.
2. A good correlation between changes in key deformation mechanisms of microballoons, such as bubble shear, burst, and fracture with *(D_avg_/h)* ratio, is established.
3. With an increasing volume fraction of bubbles, the shear angle and normalized shear stress values increased while the co-efficient of friction and friction angle reduced.
4. A key deformation mechanism was found to be a combination of bubble burst and fracture through an effective load transfer mechanism with the plastic AZ31 Mg matrix.
5. The proposed force model was in better agreement with measured results and was within 10%.

## Figures and Tables

**Table 1:** Chemical composition of matrix and reinforcement.

Chemical Composition (wt.%)						
AZ31 Mg	Al	Fe	Mn	Si	Zn	Mg
	3.10	0.005	0.25	0.02	0.73	Balance
Hollow Alumina (provided by the supplier)	Al_2_0_3_	Fe_2_0_3_	CaO	SiO_2_	Na_2_O	Avg Bubble size (mm)
	99.7	0.003	0.01	0.025	0.26	0.3–0.6

**Table 2:** Properties of matrix and reinforcement.

Matrix and Hollow Alumina Reinforcement Properties							
Material	Bulk Density (g/cm^3^)		Avg Wall Thickness (μm)	Crush Strength (MPa)	Bubble Vol%	Poisson Ratio	Thermal Conductivity (W/mK)
Hollow Alumina	1.8		0.035–0.085	125 ± 5	5%, 10%, 15%	0.231	1.5
**Mg Matrix**	**Density (g/cm^3^)**	**Poisson Ratio**	**Thermal Conductivity (W/mK)**	**Specific Heat(J/KgK)**	**Compressive Strength (MPa)**	**Yield Strength (MPa)**	**Elastic Modulus (GPa)**
	1.77	0.35	105	1150	330	172	44

**Table 3:** Conditions for cutting tests.

Experiment Conditions		
Matrix	AZ31 Magnesium	
Reinforcement	Alumina	Micro hollow thin-walled spheres syntactic foam
Microballoon		5%, 10%, 15%
volume fraction		
Cutting speed	m/min	25, 50, 100
Undeformed chip thickness	mm	0.05, 0.1, 0.15, 0.2
Width of cut	mm	3 mm
Cutting insert Sandvik™	Insert	Coated carbide
	Rake angle	6
	Clearance angle	7
	Cutting edge radius	450 μm
	Modulus of elasticity	670 GPa
	Tool hardness	23 GPa
	Tool shear strength	3.8 GPa
	Tool yield strength	7.6 GPa
	Tool Poisson ratio	0.24

**Table 4:** Constants for AZ31 Magnesium used in this study.

Matrix	A (MPa)	B (MPa)	n	C	m	Tm (°C)
AZ31 Mg	172	559	0.46	0.045	0.29	605

## Nomenclature

A,B,C, n, m	Model constants
Tm, Tr	Melting and reference temperatures
Smax	Peak compressive stress
R_ma_	Area fraction of matrix
R_cm_	Area fraction of microbubbles/balloons
χ_y_	Matrix yield strength
Z_f_	Fracture strength of bubble wall
D_avg_	Average bubble diameter
t_w_	Average wall thickness of the bubble
E_w_	Bubble crush strength
c_mf_	Bubble volume fraction
r_par_	Average radius of the bubble
S_t_	UTS of the matrix material
	Tool rake angle
φ	Shear angle
p	Equivalent plastic strain
b	Width of cut
w	Undeformed chip thickness
¶	Chip compression ratio
STo	Tool shear strength
SPL	Specific energy for plastic deformation
C_thb_	Three-body friction
C_tb_	Two-body friction
u_thb_	Coefficient of friction
n_thb_	Normal force due to three-body rolling
Ï	Probability of bubbles engaged in abrasion
